# Changing Patients’ Treatment Preferences and Values with a Decision Aid for Type 2 Diabetes Mellitus: Results from the Treatment Arm of a Randomized Controlled Trial

**DOI:** 10.1007/s13300-018-0391-7

**Published:** 2018-03-13

**Authors:** Robert A. Bailey, Alicia C. Shillington, Qing Harshaw, Martha M. Funnell, Jeffrey VanWingen, Nananda Col

**Affiliations:** 1 Janssen Scientific Affairs, LLC, Raritan, NJ USA; 2 EPI-Q Inc, Oak Brook, IL USA; 3 Department of Learning Health Sciences, University of Michigan Medical School, Ann Arbor, MI USA; 4 Family Medicine Specialists, Grand Rapids, MI USA; 5 Five Islands Consulting, Georgetown, ME USA

**Keywords:** Antihyperglycemic medication, Patient decision aid, Patient values, Shared decision-making, Type 2 diabetes mellitus, Values clarification

## Abstract

**Introduction:**

Failure to intensify treatment for type 2 diabetes mellitus (T2DM) when indicated, or clinical inertia, is a major obstacle to achieving optimal glucose control. This study investigates the impact of a values-focused patient decision aid (PDA) for T2DM antihyperglycemic agent intensification on patient values related to domains important in decision-making and preferred treatments.

**Methods:**

Patients with poorly controlled T2DM who were taking a metformin-containing regimen were recruited through physicians to access a PDA presenting evidence-based information on T2DM and antihyperglycemic agent class options. Participants’ preferences for treatment, decision-making, and the relative importance they placed on various values related to treatment options (e.g., dosing, weight gain, side effects) were assessed before and after interacting with the PDA. Changes from baseline were calculated (post-PDA minus pre-PDA difference) and assessed in univariate generalized linear models exploring associations with patients’ personal values.

**Results:**

Analyses included 114 diverse patients from 27 clinics across the US. The importance of avoiding injections, concern about hypoglycemia, and taking medications only once a day significantly decreased after interacting with the PDA [− 1.1 (*p* = 0.002), − 1.3 (*p* < 0.001), − 1.1 (*p* = 0.004), respectively], while the importance of taking medications that avoided weight gain increased [0.8 (*p* = 0.004)]. Prior to viewing the PDA, most patients (58.8%) had not begun thinking about the decision of adding a medication, and few (12.3%) indicated that they had already made a decision. Post-PDA, 46.5% could state a medication preference.

**Conclusion:**

The values-focused PDA for T2DM medication intensification prepared patients to make a shared decision with their clinician and changed patients’ values regarding what was important in making that decision. Helping patients understand their options and underlying values can promote shared decision-making and may reduce clinical inertia delaying treatment intensification.

**Funding:**

Janssen Scientific Affairs, LLC.

## Introduction

There is growing consensus that patient preferences should be incorporated into clinical decisions, but preferences can be influenced by misconceptions, fear, and personal anecdotes not applicable to an individual’s circumstances [1]. The rise of health consumerism [2, 3] has empowered patients to partner with health care providers (HCPs) to make decisions reflecting personal preferences and values. Simultaneously, consumers have greater health information access [4], although information is of variable quality and may not be relevant or specifically designed to facilitate informed decisions. Therefore, patients increasingly arrive at HCPs’ offices with preferences based on misinformation and misperceptions. Patients have difficulty applying personal values to decisions [5], yet tend to receive treatments matching initial uninformed preferences.

These issues are evident in type 2 diabetes mellitus (T2DM), a progressive disease typically requiring treatment intensification in order to maintain glycemic control [6] and prevent disease-related complications [7–10]. Most patients whose glucose levels are initially well controlled with a single drug will require intensification after 3 years [6], yet fewer than half receive additional therapy [11]. Failure to intensify when indicated, or *clinical inertia* [12], remains a major obstacle to achieving glucose control [13]. Clinical inertia is attributed to HCP knowledge, attitudes and perceptions [12, 14], and patient beliefs that insulin leads to hypoglycemia, weight gain, and complications [15–17]. Many antihyperglycemic agents are now available in addition to insulin [18]; these agents differ in their effectiveness, side effects, weight impact, route, and administration frequency. While metformin is often the initial treatment [19], the ideal sequence of drugs after metformin is no longer effective has not been definitively established and depends on how patients balance the pros and cons of treatment. This involves making difficult trade-offs. The number of options complicates decisions and adds to clinical inertia. Clinicians and patients tend to avoid decisions involving difficult trade-offs and may select inferior options to minimize the stress of choosing between two superior options [20]. Because patients value benefits and risks differently from their HCPs, decisions should include patients’ informed values. Many studies seeking to better understand preferences use hypothetical choice scenarios not directly relevant to the subjects [21, 22] or involve highly select patients who bear little resemblance to those typically seen in primary care based upon demographics, disease characteristics, and cultural risk factors.

A previously developed interactive online patient decision aid (PDA) helps patients choose which treatment to add to metformin by helping to identify and map values and preferences to treatment benefits and risks. In order to understand how PDAs influence values and preferences, we conducted a pragmatic study in nonacademic settings. The Diabetes Decision Aid for T2DM was associated with improved knowledge and self-efficacy as well as reduced decisional conflict among highly diverse US primary care patients with diabetes who interacted with the PDA versus a control group [23]. Our objectives for this analysis were to examine the values and preferences of diverse real-world patients with poorly controlled T2DM among those interacting with the PDA, and to explore how the PDA changed those values and preferences.

### Terminology

*Patient decision aids* are educational tools designed to facilitate treatment decisions in collaboration with clinicians, promoting shared decision-making [24]. *Preferences* refers to an inclination toward or away from specific options and *values* refers to what matters to a person [25].

## Methods

### Procedures

This study is part of a larger pragmatic randomized controlled trial (ClinicalTrials.gov identifier: NCT02110979) validating an interactive online PDA for T2DM during the course of routine clinical care in 27 US primary care or endocrinology clinics. Study procedures were approved by the New England Institutional Review Board. Details regarding PDA development [26] and the validation trial, including the detailed methodology, are reported elsewhere [23]. Study participants were referred by participating providers identified through two nationwide email lists [27, 28]. Primary care physicians and endocrinologists were enrolled if they managed 10 or more patients with T2DM weekly, were not academically affiliated, had access to electronic medical records or laboratory data, and had clinical staff support facilitating subject identification.

### Participants

Participants were English-speaking adults with T2DM who were taking metformin and were advised to consider additional antihyperglycemic medication to improve glycemic control. Subjects were further required to have a valid email address, access to the internet via a personal computer, and the ability to complete surveys online. Excluded were pregnant women, clinical trial participants, subjects already taking two or more medications in addition to metformin, and those with a lifetime exposure to more than three antihyperglycemic agents. This analysis examines a subset of patients who were randomized to view the PDA versus the control arm of the validation study, in order to determine the impact of the PDA on values important in medication decision-making.

### Study Intervention

**Fig. 1:**
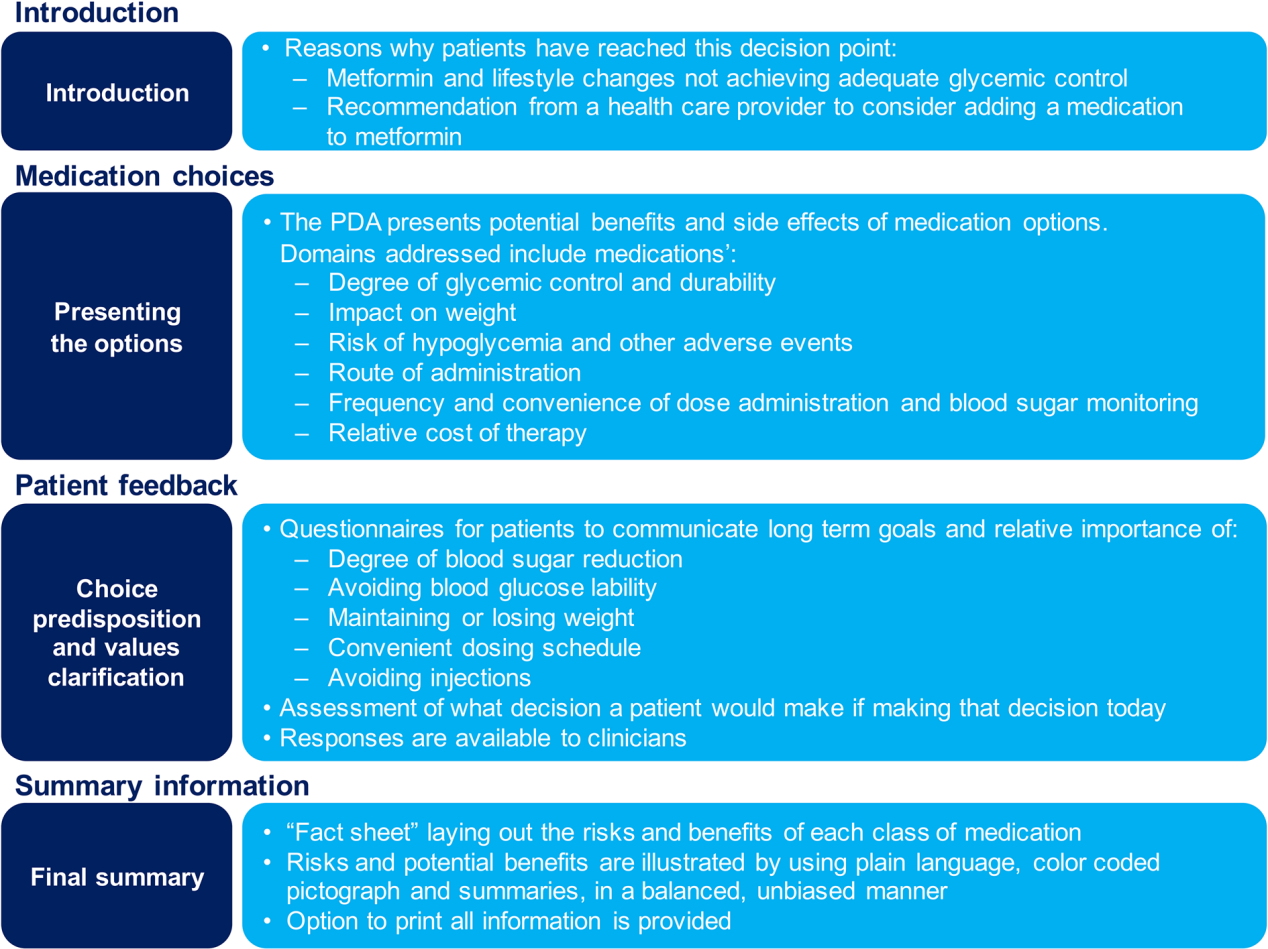
Summary of PDA content. Republished with the permission of Dove Press from [26], © 2015; permission conveyed through Copyright Clearance Center, Inc.

The interactive online Diabetes Decision Aid for T2DM addresses decisions about adding additional therapy to metformin due to poor glycemic control. The PDA content was designed to be delivered online, either in the clinical setting or at a place and time of the patient’s choosing, to prepare for a clinical consultation. Content was developed and validated to help patients understand the natural history of T2DM, the treatment options available, and the relevant risks and benefits of each option. It takes patients through a values clarification exercise in order to help them understand preferences for treatment. The PDA emphasizes values and preferences by organizing information on T2DM and treatments according to key values domains and distinguishing treatment options, including: (1) effectiveness at glycemic control; (2) weight impact; (3) hypoglycemia risk and other side effects; (4) administration route (oral versus injection); (5) treatment convenience (including dosing frequency and blood glucose monitoring); and (6) cost. Domains for inclusion in the PDA were elicited based upon input derived from patients with diabetes through a series of focus groups and a review of the literature. For each domain, the PDA compares treatment benefits and asks subjects to explore preferences and values. A summary “fact sheet” compares each medication class in relation to these domains using voiceover descriptions and simple graphics (Fig. 1).

### Outcomes and Data Collected

Study data were self-reported and collected online before interacting with the PDA (baseline), immediately after interacting with the PDA (post-PDA), and at a follow-up of 4 to 6 weeks. We assessed the patients’ readiness to engage in decision-making using the validated Stage of Decision Making scale [29], and the preferred decision role using the Control Preferences Scale [30]. The *Decision Self-Efficacy Scale* [31, 32] measured self-confidence in decision-making abilities.

#### Personal Values for Diabetes Treatment Domains

Subjects rated the importance of the following domains at baseline using a 1–10 scale (“not at all” to “extremely” important) and selected the most important of the following: “managing blood sugar to a goal;” “taking a medication that might help lose weight or not cause weight gain;” “avoiding hypoglycemia (a ‘low’);” “avoiding side-effects such as pancreatitis, fractures, urinary tract infections, and yeast infection;” “treatment costs;” “avoiding injections;” and “dosing convenience (i.e., taking medication more than once a day).” Immediately post-PDA, a subset of domains associated with clinical inertia were repeated (“importance of avoiding weight gain,” “avoiding hypoglycemia,” “avoiding injections,” and “taking medication only once a day”).^1^ *Treatment preferences* were assessed post-PDA with drop-down menus asking “…which of these medications sounds like it fits best with what you want and don’t want out of treatment?”

#### Sociodemographic and Clinical Covariates

Participants reported their age, gender, race/ethnicity, education years, height, weight, and years since T2DM diagnosis. Body mass index (BMI) was classified as < 24.9, 25–29.9, 30–39.9, and 40+ kg/m^2^ [33].

### Statistical Analyses

Data were summarized using standard descriptive statistics (means [standard deviation (SD)] for continuous variables, proportions for discrete variables). Chi-square statistics and *p* values tested for independence of observed frequencies in contingency tables. Changes in personal values were calculated by subtracting post-PDA from pre-PDA scores and comparing differences using the Wilcoxon signed rank test for skewed data.

Personal value associations at baseline were identified by developing univariate generalized linear models (GLM) for each value, using the baseline value (1–10) as the dependent variable. Independent variables included sociodemographics (age, gender, race, education), clinical variables (years since T2DM diagnosis, BMI), and decision process measures (decision self-efficacy, stage of decision-making).

Logistic regression models identified medication preference associations post-PDA. Covariates included those of theoretical interest (age, gender, BMI, decision-making stage, employment, insurance status, educational level, race/ethnicity). Variables were retained using thresholds of *p* < 0.1. To simplify interpretation, we report Exp(B) (analogous to an odds ratio) and 95% confidence interval (CI) values. We assessed goodness-of-fit using the –2 log likelihood statistic [34]. Analyses were performed using SAS^©^ [35].

### Compliance with Ethics Guidelines

All procedures performed in studies involving human participants were in accordance with the ethical standards of the institutional and/or national research committee and with the 1964 Helsinki Declaration and its later amendments or comparable ethical standards. Informed consent was obtained from all individual participants included in the study.

## Results

**Fig. 2:**
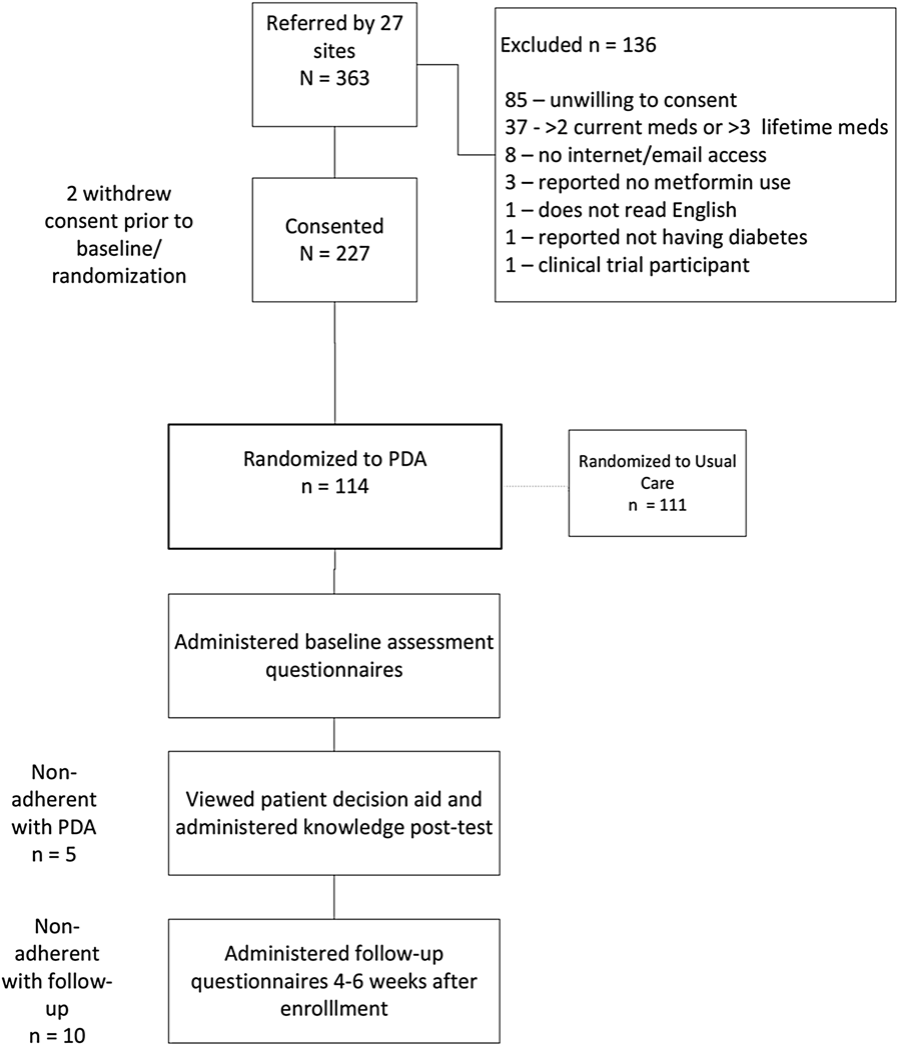
Subject disposition

One hundred fourteen diverse subjects completed post-PDA evaluations (Fig. 2). Most were diagnosed with T2DM for several years and had not begun considering choices for medications. Most preferred an active role in health care decision-making as measured by the Control Preferences Scale (Table 1). More women than men expressed a preference for shared decision-making (51.6% vs 36.5%); however, due to the small sample size, this was not statistically significant.

**Table 1:** Baseline characteristics (before viewing the PDA)

Characteristics	*N* = 114
Age, mean (SD), years Range	53.0 (13.8) 18–78
< 50 years, *n* (%)	42 (36.8)
50–64 years, *n* (%)	49 (43.0)
65+ years, *n* (%)	23 (20.2)
Male gender, *n* (%)	52 (45.6)
Race/ethnicity, *n* (%)	
White/Caucasian	53 (46.5)
African American	32 (28.1)
Hispanic	14 (12.3)
Asian/Pacific Islander	5 (4.4)
Multiracial/other	10 (8.8)
Years since diagnosis, mean (SD) Range	6.8 (6.0) < 1–30
BMI, *n* (%)	
< 24.9 kg/m^2^	12 (10.5)
25–29.9 kg/m^2^	22 (19.3)
30–39.9 kg/m^2^	48 (42.1)
40+ kg/m^2^	32 (28.1)
Education, *n* (%)	
Grade school/high school	43 (36.0)
Some college	30 (26.3)
College graduate, graduate school	41 (36.0)

Decision-making Processes	
Stage of decision-making, *n* (%)	
Haven’t begun to think about the choices	37 (32.5)
Haven’t begun to think about the choices, but interested in doing so	30 (26.3)
Considering the options	18 (15.8)
Close to selecting an option	15 (13.2)
Have already made a decision/made a decision and unlikely to change my mind	14 (12.3)
Decision control preference, *n* (%)	
Independent	29 (25.4)
Doctor	34 (29.8)
Shared	51 (44.7)
Decision self-efficacy, mean (SD) Range	85.9 (15.6) 25–100

*SD* standard deviation, *BMI* body mass index

**Fig. 3:**
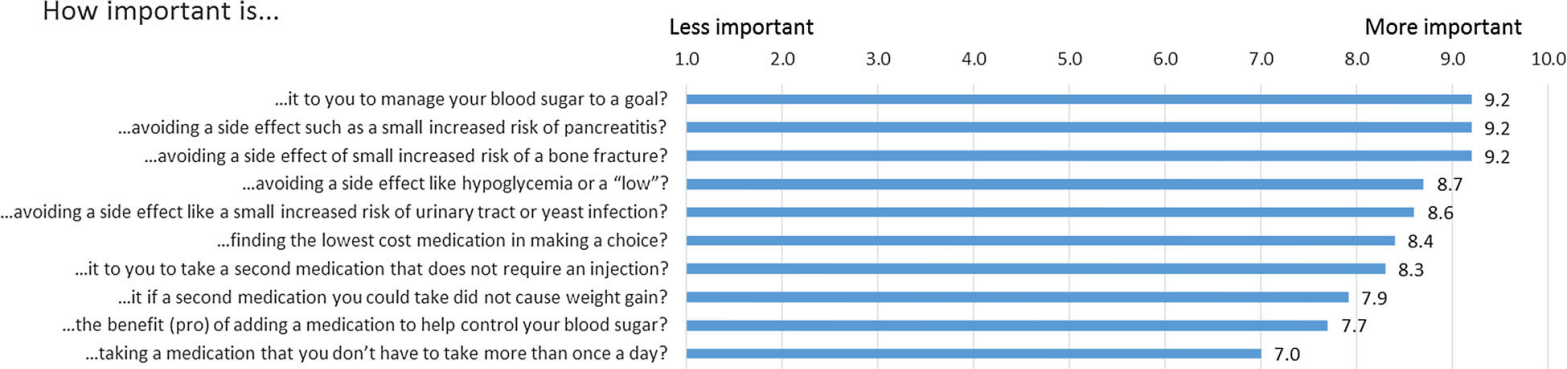
Personal values prior to viewing the PDA

Prior to viewing the PDA, the most important personal values were managing blood sugar to a goal and avoiding side effects (i.e., small increased risks of pancreatitis and bone fracture). The least important personal values were taking a medication more than once a day, adding medication, and avoiding weight gain (Fig. 3).

**Fig. 4:**
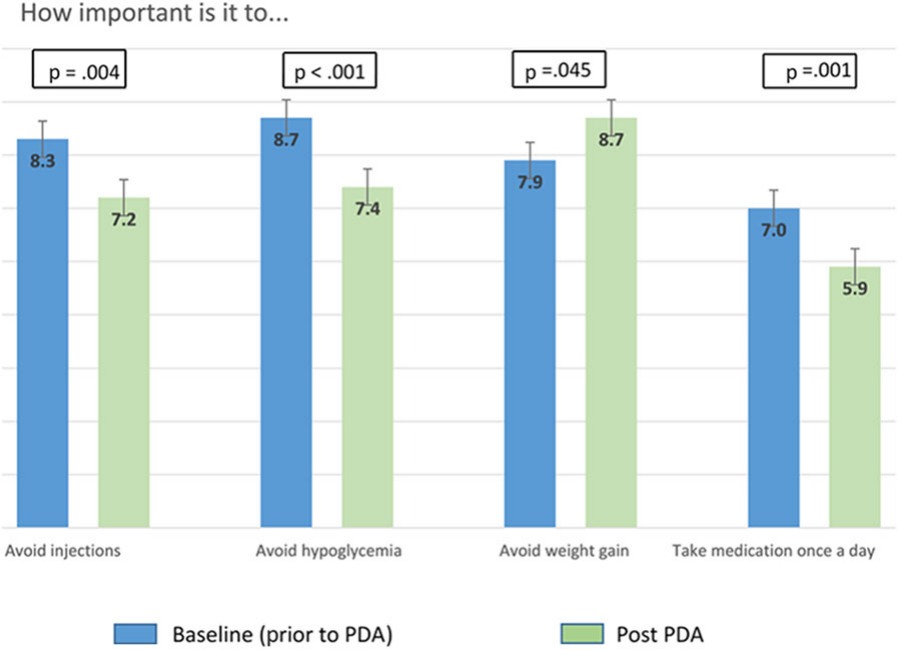
Change in select value scores after interacting with the PDA

Obesity was the sole factor associated with reporting avoidance of weight gain as a baseline value [odds ratio (OR) = 4.66; 95% CI 1.57, 13.84]. Compared with higher-educated counterparts at baseline, patients with high school diplomas or less were more likely to value avoiding hypoglycemia (OR = 2.55; 95% CI 1.13, 5.74). Compared with Caucasians, Latinos were also more likely to value avoiding hypoglycemia (OR = 4.59; 95% CI 1.63, 12.98). Our models did not identify significant associations with valuing avoiding injections or medications taken more than once a day. Post-PDA, participants placed more importance on avoiding weight gain and less importance on avoiding hypoglycemia, avoiding injections, and taking medication only once daily (Fig. 4) compared to baseline. Avoiding weight gain was the most frequently reported “biggest concern” (reported by 76.1% of patients), followed by avoiding hypoglycemia (55.6%), avoiding injections (32.1%), and taking medication only once daily (19.8%). Decisional self-efficacy increased (improved) significantly from baseline to final follow-up, from 85.9 (SD 15.6) to 89.6 (SD 12.4; *p* < 0.01).

Before interacting with the PDA, 12.3% reported already having made a decision. After seeing the PDA, 46.5% were able to indicate a drug class preference. In logistic regression analysis, decisional self-efficacy measured after the PDA was the sole parameter associated with expressing a preference (OR = 1.06, 95% CI 1.0, 1.1). Baseline decision-making stage was not significantly associated with expressing a medication preference; nor was increasing age, age, gender, BMI, employment, insurance status, educational level, or race/ethnicity.

Post-PDA, avoiding taking medication more than once daily was more important to African Americans than Caucasians and Hispanics (OR 5.31, 95% CI 1.36, 20.79). When measured after interacting with the PDA, avoiding weight gain was no longer associated with baseline BMI, and was less important to Hispanics (OR 3.78; 95% CI 1.31, 10.96) than other racial groups and the lesser educated (OR 2.25; 95% CI 1.11, 5.73) than the more educated. We could not identify any significant associations with avoiding injections post-PDA.

## Discussion

Guidelines recommend personalizing care when intensifying T2DM treatment for patients in whom metformin alone is ineffective. There is substantial scientific evidence of benefits arising from intensifying treatment in these patients [36]. However, clinical inertia is a major factor limiting implementation, at least partially due to physicians’ and patients’ misunderstandings regarding the importance of underlying values related to therapy goals and treatment [18, 37]. Six-month intensification rates in poorly controlled T2DM patients are as low as 26–37% [38, 39].

This study suggests an approach to overcome clinical inertia by helping to reshape and inform preferences through the use of a PDA. Although based on a small sample size, our findings demonstrate substantial fluidity in treatment preferences and the values influencing decisions, even among people with strong initial preferences. Over the short period of time it took to view the PDA, a large proportion changed their perceptions of their treatment decision readiness and their most-valued features of treatment. Changes in values are consistent with gaining a deeper understanding of factors influencing long-term health outcomes, and may reflect an increased willingness to sacrifice short-term difficulties (e.g., injections, hypoglycemia, dosing inconvenience) in exchange for better outcomes. While writing a prescription in response to initial stated preferences is a time-efficient approach that improves the physician’s performance metrics,^2^ it could derail shared decision-making and result in poor-quality decisions if those preferences are misinformed.

PDAs for T2DM have been shown to improve “soft” decision processes (e.g., expectations, autonomy, trust, decision-making engagement, knowledge, risk perceptions, goal documentation) [40–44]. However, the role of PDAs in improving “hard” clinical outcomes, including glycemic control, has not been confirmed in prospective studies [45–48]. Simplifying decisions about which treatment to add to metformin by eliciting and communicating informed preferences related to values associated with clinical inertia—which was the goal of this PDA—should reduce clinical inertia, resulting in more patients receiving appropriate treatment and presumably better clinical outcomes.

We could not identify an association of changing values, reinforcing the importance of values clarification followed by asking preferences. Unlike previous studies [49–51], we found no differences between older and younger subjects’ preferences for taking an active role in decisions. This suggests we should not overlook the importance of values clarification and shared decision-making with elderly patients.

Several findings were unexpected. Most participants reported a desire for shared or independent decision-making but had not prepared themselves for decision-making, suggesting that some may be unaware of the need to prepare or may not know how to access the requisite information. Baseline treatment decision readiness was not associated with treatment preference post-PDA,^3^ and many patients who initially reported making a decision were subsequently unable to choose a treatment. Perhaps patients who were unaware of the complexity of the decision shed preconceived ideas and considered more options after becoming informed (i.e., becoming less concerned about avoiding injections leads to new treatment options). *Premature diagnostic closure* is a common decision-making error in which physicians make diagnoses before considering reasonable alternatives [52]. We may be observing a similar error (*premature decision closure*) where patients select treatment before considering reasonable alternatives.

Based upon the results of this study, we make the following observations relevant to clinical care. For patients, treatment choice decisions are difficult and involve trade-offs between desired outcomes and what one is willing to sacrifice. In complex situations, it is unrealistic to expect people to know which personal values are most relevant as it relates to trade-offs. We observed that values change in response to a decision aid. Particularly, patients who do not consider weight to be as important in decision-making may especially benefit from this PDA, which increased the patients’ perceived importance of managing weight. Also, a patient initially stating which treatment they want should trigger a conversation about the decision, rather than a prescription. It may be best not to act upon a patient’s potentially misinformed initial preferences. Rather, ask how they arrived at those preferences and the options they considered. Inform patients that many people change their treatment preferences and values after learning more about the options and factors to consider, and recommend that they view a PDA before choosing a treatment.

Understanding patients’ informed preferences and values as well as enhancing decisional self-efficacy may remove barriers to care and clinical inertia. Patients who say they want to be involved in decision-making may be unaware of how to prepare and may benefit from guidance on specific actions they can take to improve health and quality information. In time-constrained clinical environments, PDAs can help to develop informed preferences and apply values, and can aid in the selection of a values-congruent treatment. Since nearly half of the patients were confident enough to express a preference after viewing the PDA, the PDA could potentially improve the efficiency of clinical consultations by helping to explain the condition and treatment options beforehand. Patients who are clearer about values and how to apply them should be better able to share with HCPs and to receive a treatment consistent with informed preferences and treatment goals [53–55].

Our study has limitations. We were unable to determine which treatment was ultimately chosen or prescribed. However, preferences strongly influence treatment decisions. We do not know whether the preferences elicited were durable. To minimize respondent burden, we limited the number of values included in our assessment, and may have missed some domains. Unmeasured, unobserved factors may contribute to decision-making beyond what we assessed. Because we limited our sample to people using the internet, our results may not be generalizable. However, the number of people from all sociodemographic groups using the internet continues to grow [56], as do public access opportunities for those with socioeconomic barriers. Benefits of making the PDA available over the internet include immediate broad access and the ability to update content. Analyses lacked a randomized control group and compared participants’ responses before and after exposure to the PDA. There is recognition that alternatives to the RCT may be more suitable for some studies.

### Unanswered Future Questions

Future studies should focus on (1) the impact of values-focused PDAs on patient–provider communication, glycemic control, clinical inertia, medication adherence, and costs, as well as (2) approaches that help clinicians to assess patients’ initial treatment preferences and values, whether they are adequately informed, and communication pointers that guide conversations about treatment for those with poorly informed preferences or values.

## Conclusions

This study revealed that a novel interactive PDA changed the preferences and values of real-world subjects with T2DM who were facing a common, difficult treatment decision with important health consequences.

## Footnotes

1 Using a 5-point Likert scale, which was later converted to a 10-point scale to allow for comparisons between assessments.

2 The Consumer Assessment of Healthcare Providers and Systems.

3 Although not statistically significant, those who reported having made a decision prior to viewing the PDA were the least likely to have a treatment preference after viewing the PDA.
